# Evolution of Methods for the Oxidation of Primary Alcohols to Carboxylic Acids: From Metal Oxides to Biocatalysis

**DOI:** 10.1021/jacsau.5c01452

**Published:** 2026-02-02

**Authors:** Jonas Spang, Francesco Mascia, Wolfgang Kroutil

**Affiliations:** † Austrian Centre of Industrial Biotechnology, Institute of Chemistry, 27267 University of Graz, Krenngasse 37, 8010 Graz, Austria; ∥ Institute of Chemistry, 27267 University of Graz, 8010 Graz, Austria; § Field of Excellence BioHealth, BioTechMed Graz, Cluster of Excellence Circular Bioengineering, University of Graz, 8010 Graz, Austria

**Keywords:** Oxidation, Primary Alcohols, Carboxylic Acids, Metal Oxides, Chemocatalysis, Biocatalysis

## Abstract

Carboxylic acids are central building blocks in fine chemical synthesis. Although the direct oxidation of primary alcohols to carboxylic acids appears straightforward from a retrosynthetic perspective, it is often associated with significant challenges. In this Perspective, we discuss concepts of one-pot approaches for the oxidation of primary alcohols to carboxylic acids. These approaches are grouped into chemical and biocatalytic concepts, which are structured according to the stoichiometric oxidant employed. The evolution of these methods is traced from traditional chemical oxidations to modern catalytic and biocatalytic strategies, underscoring the parallel shift in oxidant selection and the resulting improvements in selectivity, practical utility, and sustainability.

## Graphical abstract

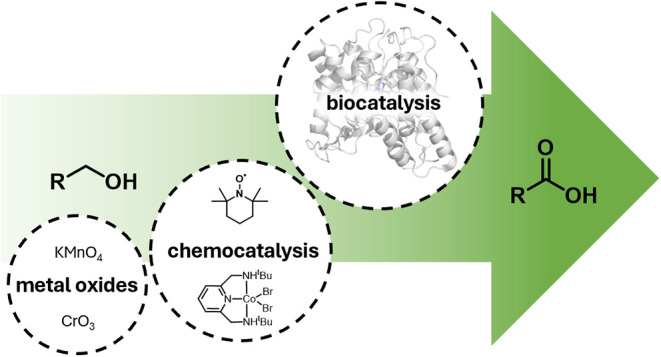

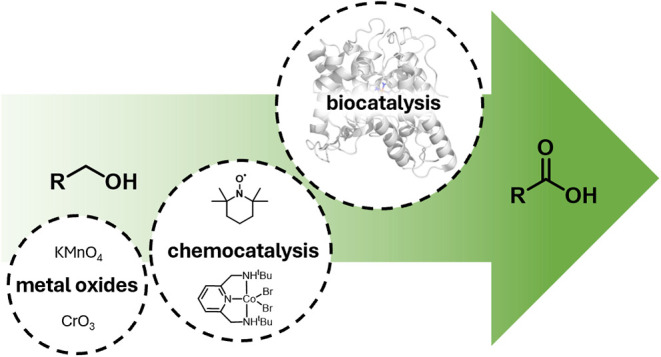

## Introduction

Carboxylic acids are ubiquitous structural motifs in chemical synthesis and represent an essential functional group in bulk and fine chemicals. For instance, over 450 pharmaceuticals contain carboxylic acid moieties, including antibiotics, anticoagulants, lipid-lowering agents, and nonsteroidal anti-inflammatory drugs (NSAIDs).^1^ A survey of GMP-scale reactions revealed that carboxylic acids were obtained preferentially by interconversion of carboxylic acid derivatives (e.g., esters, nitriles), which represented the single most common class of reactions (∼26%). In contrast, direct oxidation of primary alcohols was underrepresented,^2^ thus, the seemingly intuitive strategy of oxidizing primary alcohols to carboxylic acids is often considered a synthetic challenge, especially on an industrial scale. Therefore, instead of relying on primary alcohol oxidation, carboxylic acids are generally obtained by hydrolyzing esters or nitriles, since these routes are often considered safer, cleaner, and more robust compared to oxidation reactions. The reluctance to oxidize primary alcohols likely stems from the fact that many conventional protocols rely on nonchemoselective reagents and harsh conditions. These reagents and conditions often generate side products and significant amounts of waste.^3^ To address these issues, catalytic methods have emerged in organic synthesis to improve selectivity, efficiency, and sustainability.^4^ In parallel, rapid developments in biocatalysis have enabled complementary routes allowing oxidations under mild and potentially environmentally benign conditions.^5–14^ Here we provide a systematic overview and evaluation of chemical and biocatalytic concepts for the oxidation of primary alcohols to carboxylic acids. In both cases, the methods are grouped according to the stoichiometric oxidant employed, and for the biocatalytic section, the type of enzyme used is also considered.

## Chemical Oxidation Methods

In general, the oxidation of primary alcohols to carboxylic acids proceeds via a two-step process involving an aldehyde intermediate (Figure 1). From a thermodynamic point of view, the oxidation of a primary alcohol is exergonic (Δ*G* < 0) across a wide pH range. The driving force increases significantly at higher pH values, as the reaction is a proton-coupled electron transfer, and depending on the pH, the formation of a resonance-stabilized carboxylate further may drive the reaction.^4,15^ However, heating is often required to overcome the kinetic barrier associated with the initial oxidation step. Almost all methods rely on the presence of water, which is needed for the equilibrium between the aldehyde and the corresponding hydrate, as the latter intermediate is actually oxidized to the acid. Among selected classical chemical oxidation methods, we include examples ranging from noncatalytic processes with metalate oxidants to catalytic approaches using organic or inorganic oxidants (ideally, benign ones such as hydrogen peroxide or molecular oxygen), electrochemical systems, and dehydrogenation strategies.

**Figure 1:**
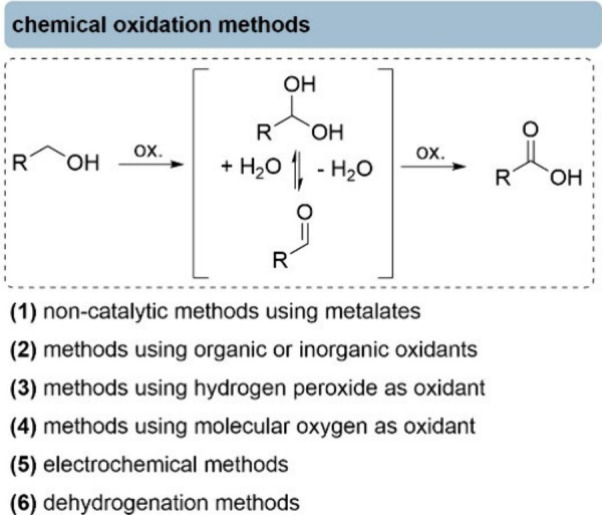
Chemical methods for the oxidation of primary alcohols to the corresponding carboxylic acids.

### Metalates

Noncatalytic oxidation methods employing stoichiometric amounts of (oxo)­metalates, such as Cr and Mn oxides, have been applied not only on a laboratory scale but also for some industrial applications. This is not surprising, as first-row transition metals in their higher oxidation states act as powerful oxidizing agents, as also detailed in a recent review.^4^

The Jones oxidation^16^ is the most prominent example of chromium-based oxidation (Figure 2a). In practice, the “Jones reagent”, prepared from chromium­(VI) trioxide in concentrated sulfuric acid, is typically used as an aqueous acetone solution. Under many discussed mechanisms, the chromium­(VI) trioxide (“Jones reagent”) reacts with the primary alcohol substrate, forming a Cr­(VI)-alkoxide ester intermediate,^17^ which undergoes E2-type β-elimination, cleaving the C–H bond in the rate-determining step.^18^ Jones oxidation typically proceeds with reasonable selectivity, since both steps, the oxidation of the primary alcohol and of the aldehyde hydrate intermediate, proceed via the ester-mediated C–H cleavage. For instance, propargylic^19^ or higher functionalized chiral alcohols^20^ can be oxidized to carboxylic acids, achieving medium to high yields.

**Figure 2:**
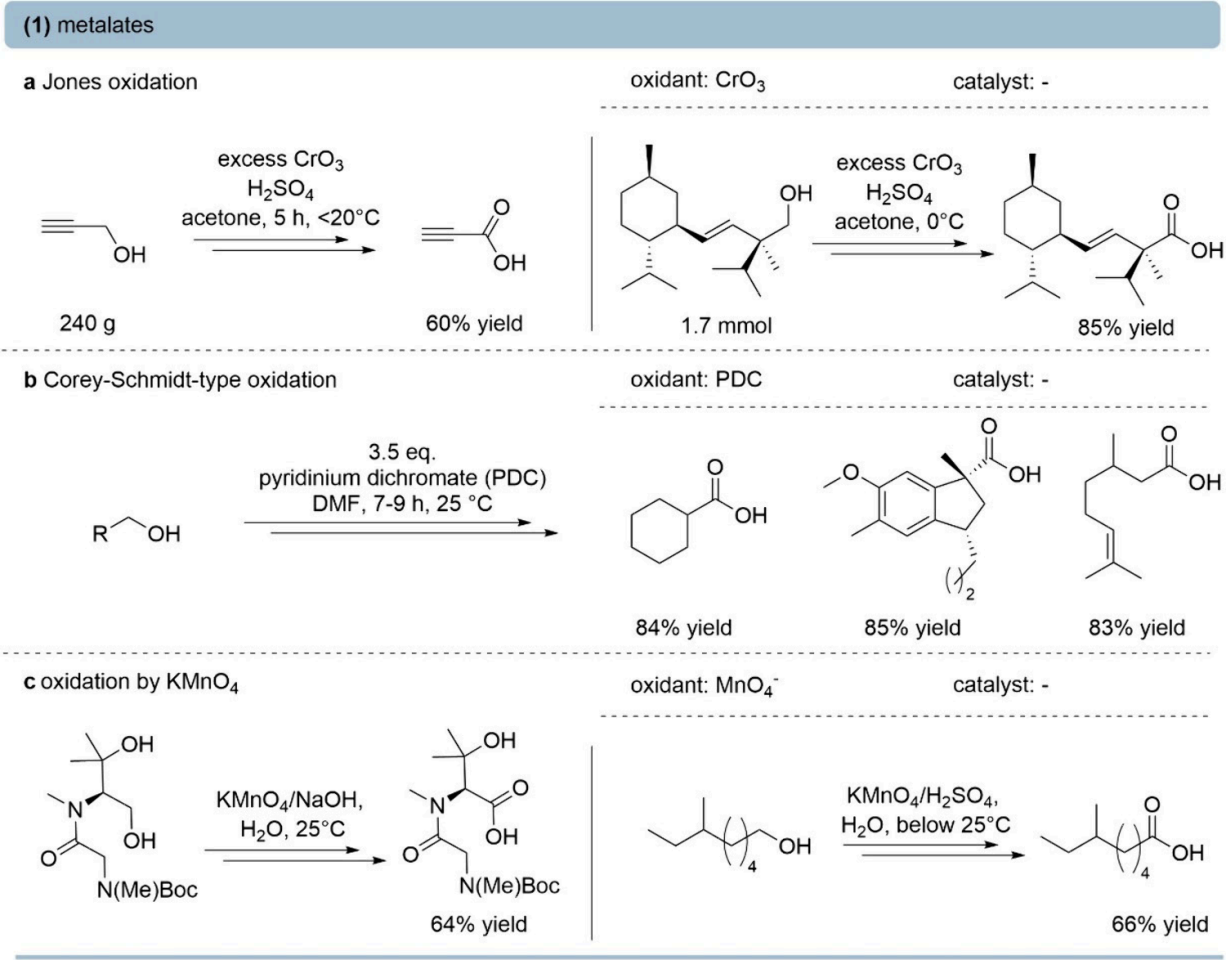
Oxidation of primary alcohols to the corresponding acids using selected (oxo)­metalates. (a) Jones oxidation employing the Jones reagent^16^ for the selective oxidation of propargylic^19^ or chiral alcohols.^20^ (b) Pyridinium dichromate (PDC) oxidation, commonly referred to as the Corey–Schmidt-type oxidation.^21^ (c) KMnO_4_ oxidation of primary alcohols under basic^23^ or acidic^25^ conditions.

The classical Corey–Schmidt oxidation typically converts primary alcohols to aldehydes; however, adjustment of reaction conditions allows access to the corresponding carboxylic acids (Figure 2b). Corey–Schmidt-type oxidations using chromium­(VI) trioxide conducted in anhydrous or wet pyridine/DMF at ambient temperature,^3^ generate pyridinium dichromate (PDC), which efficiently oxidizes nonallylic or nonbenzylic primary alcohols to carboxylic acids.^21^ However, both the Jones and Corey–Schmidt-type oxidations typically rely on toxic chromium­(VI)–oxo species, leading to stoichiometric quantities of chromium waste and often suffering from the occurrence of unspecific side reactions, as well as dimeric esters.^3^ Nevertheless, the Jones oxidation remains a robust and often high-yielding method that is frequently used in total synthesis.^22^

In addition to chromium chemistry, permanganate­(VII)-based methods are often used for the conversion of primary alcohols to carboxylic acids (Figure 2c). Permanganate oxidations under basic conditions^23^ proceed through hydrogen atom abstraction or single-electron transfer, generating Mn–O intermediates and carbon-centered radicals.^24^ This pathway often leads to side reactions and overoxidation of other C–H bonds, making permanganate a rather unselective oxidant. The oxidations carried out in water under basic^23^ as well as sometimes acidic^25^ conditions, lead to moderate yields. Thus, poor chemoselectivity and unfavorable atom economy arising from stoichiometric MnO_2_ byproducts hinder the broader applicability of potassium permanganate-mediated oxidations.^26^

Stoichiometric oxidations using metalates are considered unattractive due to poor atom economy, high E-factors (defined as the amount of waste generated per kilogram of product), and the generation of significant amounts of hazardous waste.^27^ As a result, only a few industrial processes still rely on stoichiometric chromate oxidants, while most modern large-scale oxidations have shifted toward catalytic methods.^28^

### Organic and Inorganic Oxidants

Besides noncatalytic stoichiometric oxidations with (oxo)­metalates, several catalytic approaches have been developed to improve selectivity, efficiency, and sustainability in the oxidation of primary alcohols to carboxylic acids. The Jones-type oxidation employing catalytic amounts of CrO_3_ with periodic acid (H_5_IO_6_) as the terminal oxidant affords high yields of carboxylic acids (Figure 3a).^29^ However, as mentioned before, the broader utility of chromium-catalyzed Jones-type oxidation is limited by the generation of toxic chromium waste. Tetra-*n*-propylammonium perruthenate (TPAP) with *N*-methylmorpholine *N*-oxide (NMO) typically oxidizes primary alcohols to aldehydes under mild conditions.^30^ Reaction tuning to improve acid formation can be achieved by employing higher catalyst loading, an excess of NMO, and promoting geminal diol formation (Figure 3b).^31^

**Figure 3:**
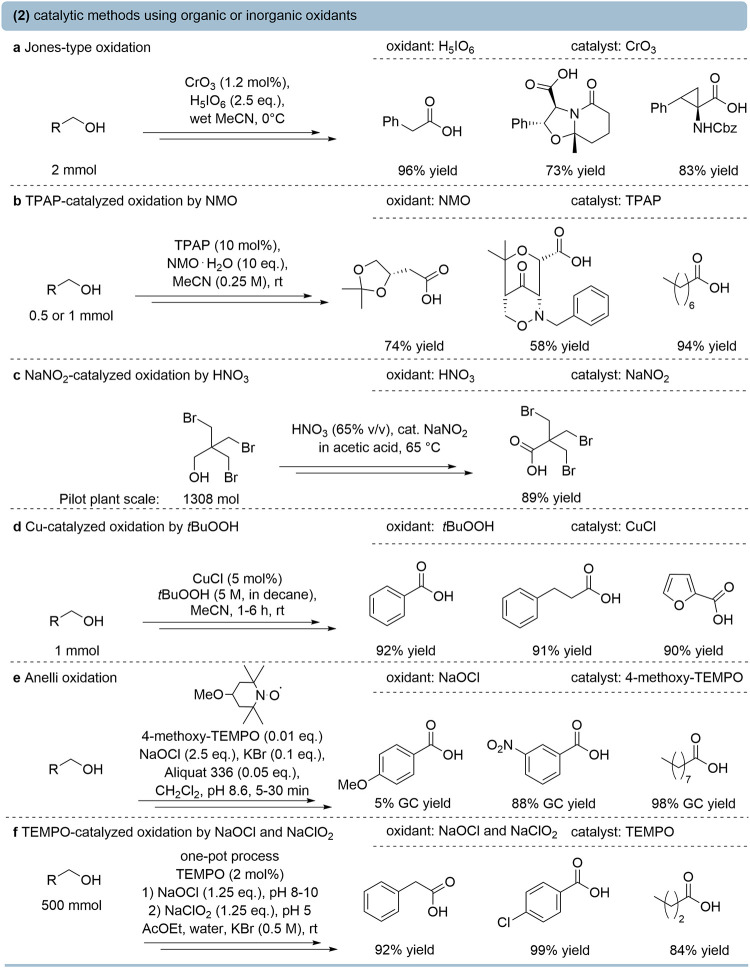
Catalytic oxidation of primary alcohols to carboxylic acids by selected organic or inorganic oxidants. (a) Jones-type oxidation of primary alcohols catalyzed by CrO_3_ using H_5_IO_6_ as a terminal oxidant.^29^ (b) Tetra*-n*-propylammonium perruthenate (TPAP)-catalyzed oxidation using *N*-methylmorpholin-*N*-oxide (NMO) as the stoichiometric oxidant.^31^ (c) Oxidation by nitric acid using sodium nitrite as the catalyst.^32,33^ (d) Cu­(I)-catalyzed oxidation using *tert*-butyl hydroperoxide (*t*BuOOH).^34^ (e) 4-Methoxy-TEMPO-catalyzed Anelli oxidation using potassium hypochlorite.^35^ (f) TEMPO-catalyzed oxidation of primary alcohols in a two-step reaction, one-pot process.^36^

Oxidation via nitric acid catalyzed by NaNO_2_ is scalable and effective for forming carboxylic acids (Figure 3c).^32,33^ However, the medium is corrosive, and the reaction selectivity is moderate, which can result in overoxidation.

Copper-catalyzed systems with *tert*-butyl hydroperoxide (*t*BuOOH) generally oxidize primary alcohols to aldehydes, but acid formation is occasionally observed under forcing conditions (e.g., 5 equiv of *t*BuOOH) (Figure 3d).^34^ The high stoichiometric amount of oxidant and waste is a drawback of this protocol.

In contrast to the methods discussed above, nitroxyl radical-mediated oxidations constitute highly efficient methodologies with proven applicability from laboratory to industrial scale. Anelli and co-workers showed that 4-methoxy-2,2,6,6-tetramethylpiperidin-1-oxyl (4-methoxy-TEMPO) in combination with aqueous NaOCl under biphasic, buffered conditions (pH 8.6) enables the selective oxidation of primary alcohols to aldehydes. TEMPO undergoes reversible oxidation to the oxoammonium cation, which is generally proposed to act as the active oxidant, abstracting a hydride (or proton-coupled electron) from the alcohol to form the aldehyde, while regenerating TEMPO via reoxidation. Notably, under otherwise identical conditions, aldehydes undergo subsequent oxidation to carboxylic acids in the presence of bromide ions and a quaternary ammonium phase-transfer catalyst (Figure 3e).^35^

A broad range of TEMPO-based methods has been developed and partially applied in industry over the past decades. For instance, a two-step, one-pot oxidation method using NaOCl and NaClO_2_ as stoichiometric oxidants was developed at Fujisawa Pharmaceutical Co., Ltd. (Figure 3f).^36^ Optimization of pH and sequential addition of the oxidant enabled multikilogram synthesis of carboxylic acids in high yields. Although partially used at a larger scale, TEMPO-mediated oxidation using NaOCl and NaClO_2_ faces challenges such as the cost of TEMPO,^37^ difficulties in catalyst recycling,^38^ and the more demanding downstream processing associated with halide-containing waste.^39^

### Hydrogen Peroxide as the Oxidant

Using hydrogen peroxide as the oxidant offers a high potential for the development of sustainable oxidation methods, as only water as a benign coproduct is generated when using suitable catalysts.^40^ Phase-transfer-catalysis systems have been introduced for this purpose, with a notable example relying on Na_2_WO_4_ (Figure 4a).^41^ Ammonium hydrogensulfate is essential for high reactivity, with the acidity of the counteranion playing a decisive role in stabilizing the peroxotungstate species and facilitating their transfer into the organic phase. Maximum activity was achieved at an initial pH of ∼2 and elevated temperatures (∼90 °C). These conditions stabilize the peroxotungstate species and accelerate the conversion of aldehydes via their hydrates to the corresponding carboxylic acids, resulting in high yields.

**Figure 4:**
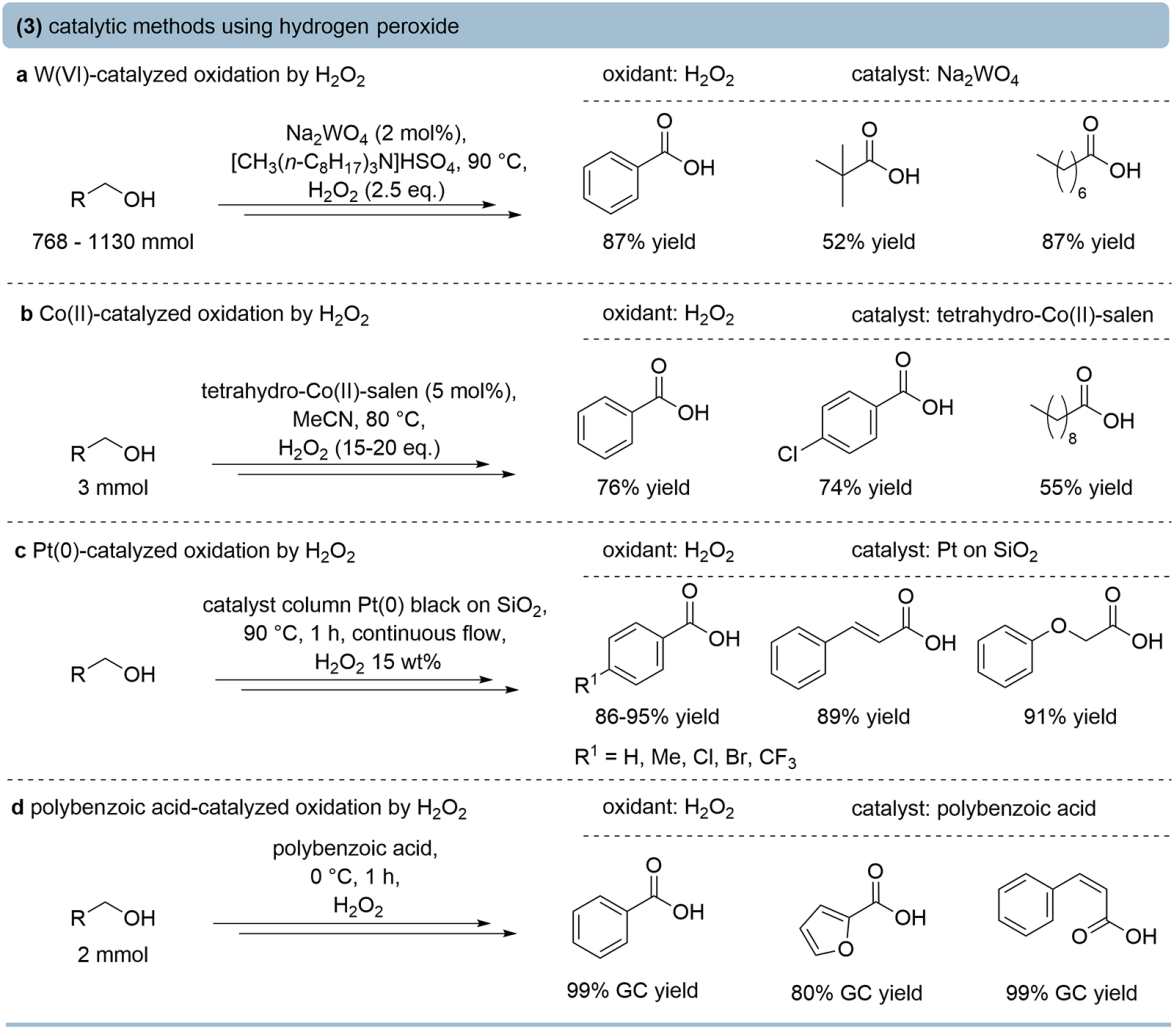
Selected examples for the oxidation of primary alcohols using H_2_O_2_ as the stoichiometric oxidant. (a) Na_2_WO_4_-catalyzed oxidation using H_2_O_2_.^41^ (b) Co­(II)-catalyzed oxidation using H_2_O_2_.^42^ (c) Pt(0) nanoparticles supported on silica catalyze the oxidation of a wide range of aliphatic, benzylic, and allylic primary alcohols to the corresponding carboxylic acids in continuous flow.^43^ (d) Polymeric peroxybenzoic acid-catalyzed primary alcohol oxidation to acids.^44^

Co­(II)–salen and related complexes efficiently catalyze the oxidation of benzylic and aliphatic primary alcohols to carboxylic acids using 30% hydrogen peroxide in acetonitrile at elevated temperatures (∼80 °C), affording good yields (Figure 4b).^42^ Furthermore, immobilized cobalt-based catalysts have been developed, with a notable example being cobalt­(II) thioporphyrazine immobilized on magnetic silica, facilitating the selective oxidation of benzylic alcohols to benzoic acids in an aqueous medium using hydrogen peroxide as the oxidant.^45^

Polyoxometalates have attracted attention as recyclable peroxo catalysts. It was demonstrated that Zn-substituted polyoxometalates promote clean alcohol-to-acid oxidations with hydrogen peroxide under microwave irradiation, highlighting the role of heteropolyacid acidity in stabilizing reactive peroxo intermediates.^46^ More recently, amino acid-modified Keggin-type polyoxometalates were shown to selectively oxidize primary aliphatic alcohols such as 1-propanol to propionic acid in aqueous media,^47^ while tungstate-based polyoxometalates immobilized on porous aromatic frameworks delivered high yields of benzoic acids under heterogeneous aqueous conditions.^48^

In parallel, noble metal nanoparticles have emerged as effective heterogeneous catalysts for hydrogen peroxide-driven oxidations. Pt(0) nanoparticles supported on silica were reported for the oxidation of a wide range of aliphatic, benzylic, and allylic primary alcohols to the corresponding carboxylic acids in continuous flow, with isolated yields reaching up to 98% (Figure 4c).^43^ In addition to transition metal catalysis, metal-free hydrogen peroxide activation strategies were developed. For instance, peroxybenzoic acid was formed in situ upon treatment of polymeric benzoic acid with hydrogen peroxide. The reactive polymeric peracid was capable of oxidizing primary alcohols to acids with high yields and selectivity (Figure 4d).^44^

Yet, oxidation of primary alcohols using hydrogen peroxide has been mainly carried out at the laboratory scale.^49^ Indeed, these routes remain a niche but can be attractive when the advantages of aqueous media, low halide and metal content, and higher safety in flow systems outweigh the issues related to handling hydrogen peroxide and potentially dangerous peroxo species.^50^ Broader application will depend on the development of robust, recyclable heterogeneous catalysts, corrosion-resistant operation conditions at pH 1–3, metered hydrogen peroxide delivery, or in situ hydrogen peroxide generation.

### Molecular Oxygen as the Oxidant

Similar to hydrogen peroxide, molecular oxygen is an oxidant of choice for potential sustainable oxidations due to its abundance, atom economy, and benign reaction coproduct (water).^51^ Historically, large-scale hydrocarbon oxidations with O_2_ enabled industrial carboxylic acid production. For instance, in 1920, early paraffin oxidation studies showed that diluted O_2_ or air streams resulted in higher fatty acid yields, although overoxidation and byproduct formation also occurred.^52^

Many catalytic methods have been developed for the activation of molecular oxygen. For instance, an Fe/TEMPO system [Fe­(NO_3_)_3_·9H_2_O/TEMPO/KCl] achieved aerobic oxidation of alcohols to carboxylic acids at room temperature (Figure 5a, left part).^53^ The Fe­(NO_3_)_3_/TEMPO catalytic system generates an oxoammonium species that oxidizes alcohols to aldehydes via β-H elimination. Subsequently, Fe-mediated hydration and further oxidation convert the aldehyde into carboxylic acid, with O_2_ serving as the terminal oxidant and NO_*x*_ cycling the Fe^2+^/Fe^3+^ redox couple. Notably, the Fe^3+^-mediated hydration of the aldehyde forms a metalated hydrate, which subsequently undergoes β-H elimination, yielding the carboxylic acid. A broad scope of primary alcohols bearing various groups like esters, ethers, halides, heterocycles, and alkynes was oxidized to the corresponding carboxylic acids, including the gram-scale synthesis of phlomic acid.

**Figure 5:**
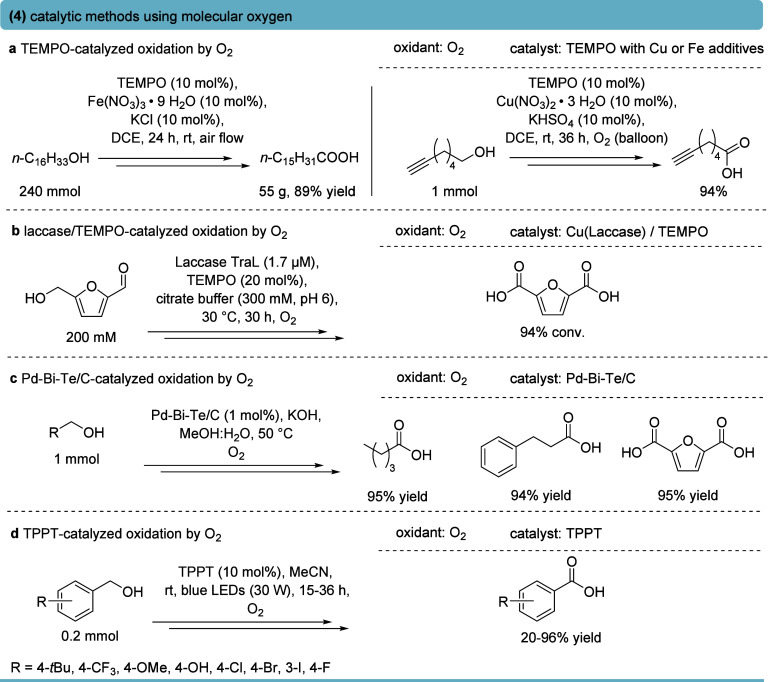
Selected examples for the oxidation of primary alcohol to the corresponding carboxylic acids employing molecular oxygen as oxidant. (a) TEMPO together with Fe- or Cu-catalyzed oxidation.^53,54^ (b) Chemoenzymatic oxidation using the TEMPO/laccase system.^56^ (c) Heterogeneous Pd–Bi–Te/C-catalyzed oxidation.^57^ (d) Photocatalytic oxidation using 2,4,6-triphenylpyrylium tetrafluoroborate (TPPT) catalyst.^58^

In a complementary method, primary alcohols were efficiently oxidized to acids employing Cu­(NO_3_)_2_·3H_2_O (10 mol %) with catalytic TEMPO and KHSO_4_ under O_2_ (Figure 5a, right part).^54^ The sulfate was shown to be essential for aldehyde hydration, consequently accelerating the overall aldehyde-to-acid step. Primary alcohols containing aliphatic, benzylic, alkynyl, amino, as well as sterically demanding groups were oxidized in high isolated yields. However, both the iron- or copper-dependent TEMPO catalytic systems require rather high catalyst loading and rely on high amounts of additives, which may reduce the overall process efficiency.

Chemoenzymatic TEMPO-based protocols were developed, with the multicopper oxidase laccase continuously regenerating the oxoammonium species, which catalyzed the primary alcohol oxidation. Whereas conventional laccase–mediator systems typically stall at the aldehyde stage,^55^ solvent engineering using citrate buffer promoted aldehyde hydration and enabled efficient conversion of 5-hydroxymethylfurfural to 2,5-furandicarboxylic acid with up to 94% yield at 200 mM substrate loading (Figure 5b).^56^ The method operates under mild aqueous conditions and is scalable, though activity remains strongly buffer-dependent, and the substrate scope was only shown for aromatic alcohols.

A Pd–Bi–Te/C heterogeneous catalyst was applied to mediate aerobic oxidation of benzylic, aliphatic, and heteroaryl primary alcohols to the corresponding carboxylic acids, operating under O_2_ at 50 °C in continuous flow (Figure 5c).^57^ Bi and Te promoters act synergistically to suppress aldehyde accumulation and inhibit Pd deactivation. Primary alcohols were oxidized to carboxylic acids with yields of >90% across a broad substrate range in continuous-flow packed-bed reactors.

A direct, metal-free alternative was introduced by employing a photoredox strategy with 2,4,6-triphenylpyrylium tetrafluoroborate (TPPT). Under blue LED irradiation, excited TPPT activates O_2_ through two orthogonal pathways. Initially, a single-electron transfer generates superoxide for the alcohol-to-aldehyde oxidation step, while energy transfer produces singlet oxygen that converts aldehydes to carboxylic acids. This dual activation enabled sequential oxidation, affording benzoic acids in up to 96% yield (Figure 5d).^58^ However, reduced oxidation efficiency for certain substituted benzyl alcohols was observed,^58^ and like many photoredox protocols, challenges in scale-up due to light penetration issues may need to be addressed. Several additional aerobic primary alcohol oxidation strategies were reported.

For instance, Au/mesoporous TiO_2_ in water converts primary alcohols to the corresponding acids using O_2_ as the sole oxidant.^59^ Furthermore, organocatalytic oxidation methods can harness molecular oxygen without metals: ethylene-bridged flavinium salts accomplished metal-free aerobic oxidation of benzylic substrates (toluene derivatives and benzyl alcohols) to benzoic acids under visible light.^60^

Despite the surge of catalytic strategies in the laboratory, large-scale carboxylic acid production continues to rely on established aerobic O_2_ routes. For instance, the AMOCO process, employing Co–Mn–Br catalysts for the liquid-phase oxidation of *p*-xylene via the corresponding primary alcohol, remains the industrial standard for terephthalic acid manufacturing, an essential platform chemical, e.g., as a monomer in polyester and PET production.^61^ Similarly, the industrial standard for benzoic acid synthesis is the Co-catalyzed aerobic oxidation of toluene via the alcohol intermediate, typically conducted in the liquid phase under elevated temperature and pressure.^62,63^ These processes underscore both the robustness and limitations of traditional autoxidation chemistry on a bulk scale. Both processes are highly efficient and scalable; however, they require harsh conditions, strong promoters, and specialized reactors to address selectivity and safety.

### Electrochemical Oxidation

In recent years, electrochemical oxidation protocols have frequently been reported.^64,65^ In general, these methods involve either a redox mediator that cycles between oxidation of the substrate and reductive recovery, or direct oxidation of primary alcohols occurring at the (activated) anode.

Ni-based (oxy)­hydroxides [e.g., Ni­(OH)_2_ anodes] remain the most studied systems, which form the active Ni species upon activation by current. Mechanistic studies revealed two potential-dependent regimes, involving either hydrogen atom transfer (lower potentials) or hydride transfer at Ni­(IV) centers (higher potentials).^66,67^ The structural state of NiOOH is decisive, with β-NiOOH (more stable, lower valence) promoting aldehyde formation, while γ-NiOOH (highly active, disordered, higher-valence state) and strongly alkaline conditions drive the complete oxidation to carboxylates.^68^ The catalytic cycle is closed by the cathodic hydrogen evolution reaction.

Oxidation of primary alkyl, aryl, and allylic alcohols at Ni­(OH)_2_ anodes was reported as early as 1979, demonstrating that in alkaline solution, primary alcohols can be oxidized to carboxylic acids (Figure 6a).^69^ Furthermore, mainly heteroaromatic primary alcohols were oxidized by a Ni­(OH)_2_-coated anode to carboxylic acids in batch and flow with minimal Ni-leaching (Figure 6b).^70^

**Figure 6:**
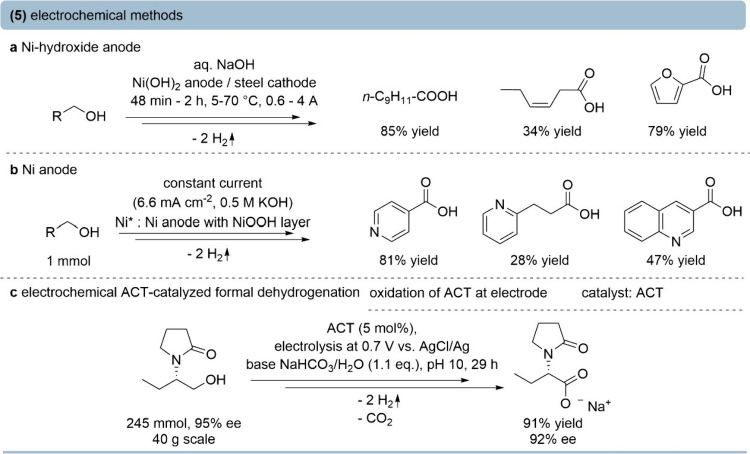
Oxidation of primary alcohols to the corresponding carboxylic acid by electrochemical methods. (a) Anodic Ni­(OH)_2_-mediated oxidation in alkaline solution.^69^ (b) Oxidation of heteroaromatic primary alcohols with a NiOOH-coated anode.^70^ (c) 4-Acetamido-TEMPO (ACT)-mediated electrochemical dehydrogenation of primary alcohols.^71^

Aminoxyl mediators such as TEMPO and 4-acetamido-TEMPO (ACT) allow milder and more functional-group-tolerant oxidations toward carboxylic acids.^71,72^ In an ACT-mediated electrochemical oxidation, alcohols and aldehydes were converted to carboxylic acids (Figure 6c).^71^ This process was carried out at room temperature in aqueous buffer, releasing H_2_ as the sole coproduct. A broad substrate scope and preserved stereochemical integrity were achieved, including gram-scale synthesis of a precursor of the pharmaceutical levetiracetam.

Both Ni­(OH)_2_-anodic and ACT-mediated electrochemical oxidation of primary alcohols represent efficient and sustainable alternatives to other conventional oxidants, offering high selectivity while generating H_2_ as the sole byproduct. However, despite these advantages, their performance is closely tied to the electrode configuration and mass transport in aqueous media, which can pose challenges for scale-up.

Additionally, alternative methods were also investigated, but mostly remain niche. For instance, the “oxidation by reduction” concept employs cathodically activated persulfate to generate sulfate radicals that oxidize alcohols.^73^ While effective, compared to true electrocatalysis, this method requires stoichiometric oxidants and produces more waste, limiting its sustainability. Furthermore, mixed oxides such as Co_2_NiO_4_ enable tuning the reaction selectivity, though the competing oxygen evolution reaction remains a limitation.^74^

### Dehydrogenation

Besides methods relying on external oxidants, conversion of primary alcohols to carboxylic acids can also be achieved via catalytic dehydrogenation, in which H_2_ is released in an “acceptorless” option or by employing a sacrificial hydrogen acceptor.^4^

A broad spectrum of acceptorless dehydrogenation methods for the oxidation of primary alcohols has been developed, especially in the field of homogeneous catalysis. For instance, Co­(II) pincer complexes catalyze the oxidation of primary alcohols to carboxylate salts with H_2_ as the only byproduct, while showing functional group tolerance and high yields (Figure 7a).^75^ Comparably, Ni­(II) N′NN′ pincer complexes catalyze acceptorless dehydrogenation of primary alcohols, resulting in 40–90% yield of the corresponding carboxylic acid (Figure 7b).^76^ Ru­(II) complexes with benzimidazole–pyridine pincers represent one of the most efficient homogeneous systems. In alcohol/CsOH medium, primary alcohols were formally oxidized to carboxylic acids in high yields with TONs reaching ∼10,000 (Figure 7c).^77^

**Figure 7:**
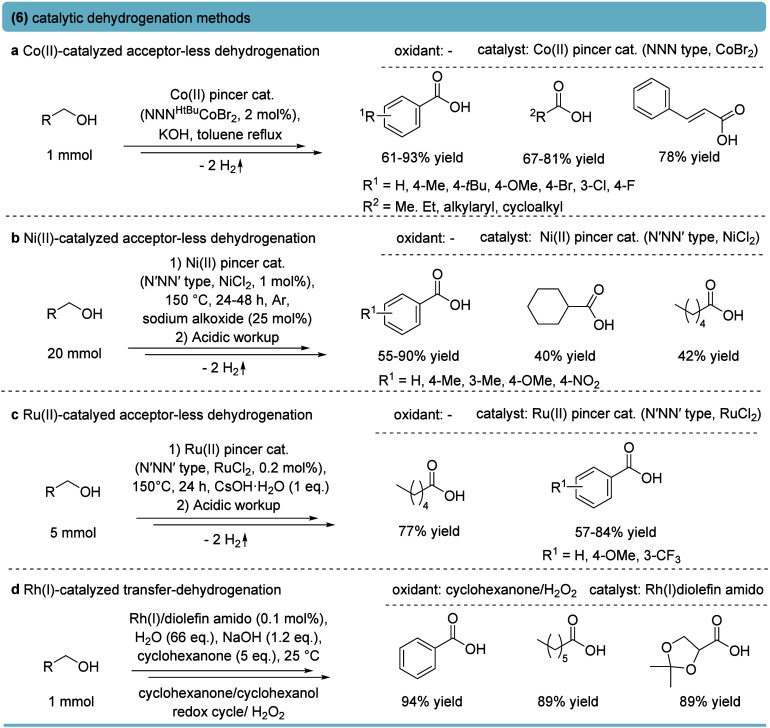
Formal oxidation of primary alcohols by catalytic acceptorless dehydrogenation or transfer dehydrogenation. (a) Co­(II)-catalyzed acceptorless dehydrogenation of primary alcohols.^75^ (b) Ni­(II)-catalyzed acceptorless dehydrogenation of primary alcohols.^76^ (c) Ru­(II)-catalyzed acceptorless dehydrogenation of primary alcohols.^77^ (d) Dehydrogenative coupling (transfer dehydrogenation) for primary alcohol oxidation catalyzed by Rh­(I) acetamido catalyst.^78^

A Rh­(I)–amido complex, [Rh­(trop_2_N)­(PPh_3_)], was reported to enable the dehydrogenative coupling of primary alcohols in the presence of water to afford carboxylic acids (Figure 7d).^78^ Although Rh­(I)–amido-catalyzed dehydrogenative coupling enabled the mild, chemoselective oxidation of primary alcohols to the carboxylic acids with recyclable cyclohexanone/H_2_O_2_ as a hydrogen shuttle, its reliance on Rh, the air sensitivity, and the system complexity pose limits to its application.

From an applicative perspective, acceptorless dehydrogenation offers advantages such as not relying on oxidants, having H_2_ as the sole coproduct, and a broad substrate spectrum across benzylic, aliphatic, and functionalized alcohols. However, these methods are often carried out under harsh reaction conditions (∼150 °C, strong bases, and extended reaction times). Ru­(II)-catalyzed systems often suffer from product precipitation, Ni­(II)-catalyzed ones from low activity and etherification side reactions, and Co­(II)-based approaches from high catalyst loadings and reduced efficiency for electron-poor substrates. Current moderate turnover numbers for bulk chemicals, operational challenges, as well as varying costs of the catalyst, e.g., Ru­(II)-based methods, represent disadvantages.

## Biocatalytic Oxidations

Although the oxidation of primary alcohols to carboxylic acids is still predominantly carried out using chemical methods, the rapid development of biocatalysis has opened new opportunities over the past decades.^6,8–12^ Biocatalysis, the use of enzymes to catalyze chemical transformations, enables precise reactivity control via finely tuned interactions in the active sites of enzymes. This allows high regio- and chemoselectivity, which minimizes or avoids side reactions. Biocatalysts typically operate in water as a solvent under ambient temperature and pressure, achieving high turnover frequencies (in general per second). Additionally, enzymes are produced from renewables and are biodegradable.

Nevertheless, biocatalysis, particularly when employing wild-type enzymes, still faces some challenges, including low to moderate substrate loadings, limited space-time yields, and moderate catalyst stability compared to chemical catalysts. To address these limitations, enzyme engineering is a powerful strategy for adapting enzymes to the desired reaction conditions.^79–83^ Typically, enzyme engineering in combination with process engineering enables efficient and scalable biocatalytic transformations.

Analogous to chemical oxidation methods, biocatalytic oxidation of primary alcohols to carboxylic acids proceeds via two reaction steps with aldehydes and the corresponding hydrates as intermediates (Figure 8a). Both oxidation steps may be catalyzed by the same enzyme or by two different enzymes, each specialized either for the alcohol or the aldehyde oxidation step. Consequently, the reaction mechanisms differ depending on the type of enzyme used and the corresponding oxidant.

**Figure 8:**
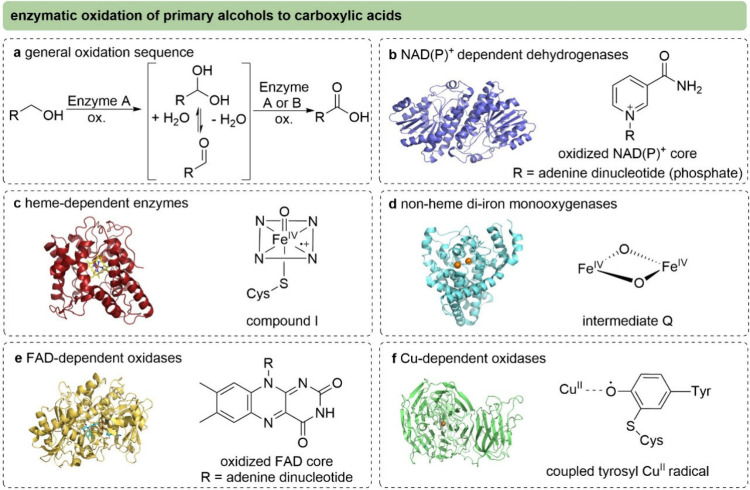
Reactive species involved in the enzymatic oxidation of primary alcohols to carboxylic acids. (a) Oxidation of primary alcohols to carboxylic acids. (b) Alcohol dehydrogenase (e.g., ADH, PDB ID 1A4U) and aldehyde dehydrogenase (AlDH) catalyze oxidations using NAD­(P)^+^ as the hydride acceptor.^84,85^ (c) Heme-containing enzymes such as CYP450s or unspecific peroxygenases (UPOs) catalyze primary alcohol oxidation via a compound I intermediate through a radical rebound mechanism (e.g., UPO, PDB ID 9J1Q).^87,89,91^ (d) Non-heme diiron monooxygenases catalyze oxidations via intermediate Q through a radical rebound-type mechanism (e.g., alkane monooxygenase, PDB ID 8F6T).^92^ (e) FAD-dependent oxidases catalyze oxidations by overall hydride abstraction, with FAD serving as the electron acceptor (e.g., choline oxidase, PDB ID 4MJW).^94^ (f) Cu-radical oxidases catalyze oxidations by hydrogen atom abstraction through a tyrosyl-Cu­(II) radical in a radical-type mechanism (e.g., galactose oxidase, PDB ID 1GOF).^99^

Alcohol or aldehyde dehydrogenases (ADH/AlDH) catalyzed oxidations involve a hydride transfer from the α-carbon of the substrate to the oxidized nicotinamide adenine dinucleotide cofactor, NAD­(P)^+^ (Figure 8b).^84,85^ While AlDHs selectively catalyze the oxidation of aldehydes to acids via a monothioacetal intermediate covalently bound to an active site cysteine,^85^ ADHs can perform either just the oxidation of the alcohol or both oxidation steps, thus alcohol oxidation and aldehyde oxidation, depending on the specific enzyme.^86^

Heme-dependent enzymes rely on a highly reactive oxoferryl porphyrin π-cation radical (compound I, Figure 8c) as the reactive species, which is formed from the resting state of heme using molecular oxygen/electrons or hydrogen peroxide. While cytochrome P450 monooxygenases (CYP450) require molecular oxygen in combination with electron shuttling,^87,88^ peroxygenases shortcut the formation of compound I by using H_2_O_2_, thereby circumventing the need for electron shuttling and O_2_.^89–91^ Compound I can abstract a hydrogen atom from the substrate, forming a carbon-centered substrate radical and an Fe­(IV)–OH species. The latter subsequently transfers a hydroxyl radical to the substrate via a radical rebound, yielding the oxidized product.^88^

Non-heme diiron monooxygenases coordinate two iron Fe­(III) atoms in their active site. Upon reduction of the two Fe­(III) to Fe­(II), O_2_ is bound; subsequent cleavage of the O–O bond leads to formation of the reactive Fe­(IV)–Fe­(IV)–O_2_ diamond-core intermediate structure (intermediate Q) (Figure 8d). The reactive species abstracts a hydrogen atom from the substrate, followed by oxygen rebound, releasing the oxidized product.^92,93^

Flavin-dependent oxidases bind flavin adenine dinucleotide (FAD) covalently or non-covalently in their active site (Figure 8e).^94–98^ Overall, a hydride is transferred from the substrate to the oxidized FAD cofactor, generating FADH_2_, which is oxidized back to FAD at the expense of molecular oxygen, leading to H_2_O_2_ as a coproduct.

Copper oxidases catalyze the aerobic oxidation of primary alcohols to carboxylic acids via a coupled metal-radical mechanism (Figure 8f). In the Cu-dependent galactose oxidase, a mononuclear Cu center ligated by histidine residues together with a Cys–Tyr radical mediates the two-electron oxidation of alcohol or aldehyde substrates. At the Cu­(I) center, O_2_ is activated and reduced to H_2_O_2_, thereby closing the catalytic cycle.^95,96,99^

The biocatalytic methods are grouped here based on the stoichiometric oxidant (organic molecules, O_2_, or H_2_O_2_), considering also the type of enzyme involved. Nevertheless, approaches using wild-type whole cells are discussed first.

### Wild-Type Whole Cells

Historically, the biocatalytic oxidation of primary alcohols to carboxylic acids started with the use of wild-type whole-cell biocatalysts in the second half of the 20th century.^100–102^ In general, the methods rely on air or O_2_-enriched air to provide molecular oxygen as an oxidant in sufficient quantity; however, also organic compounds like glucose or glycerol are required as reagents. Notably, the whole cells may be used either in a resting or fermenting state.

The most common microorganisms employed as whole-cell biocatalysts were acetic acid bacteria, such as *Acetobacter aceti*, which was used for the enantioselective synthesis of 2-phenylpropionic acid,^103^ phenylacetic acid,^104^ or the regio- and stereoselective oxidation of chiral 2-alkyl-1,3-diols to the corresponding chiral 2-hydroxymethyl alkanoic acids (Figure 9a).^105^

**Figure 9:**
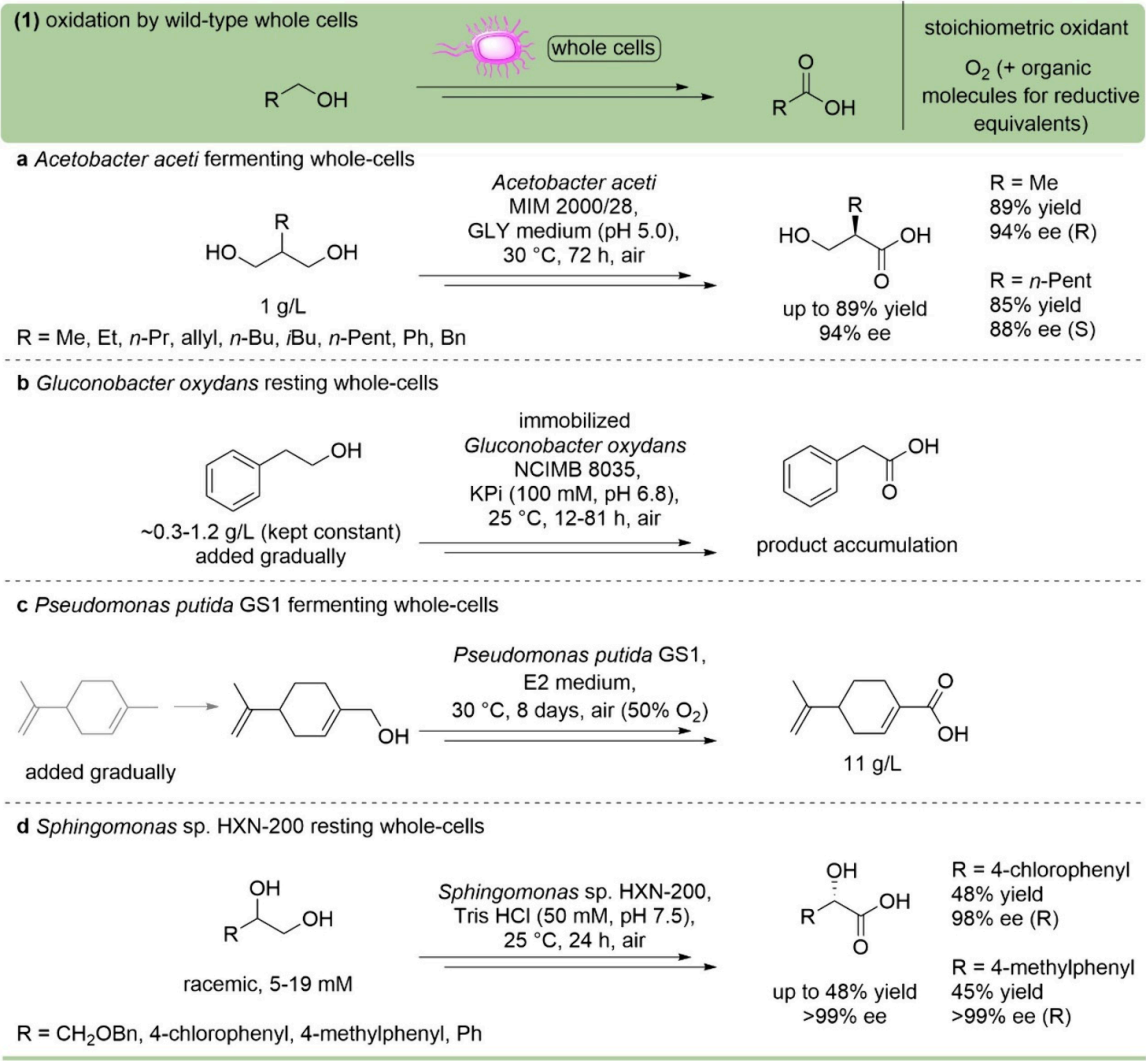
Oxidation of primary alcohols with wild-type whole-cell biocatalysts. (a) Multistep oxidation of 1,3-diols catalyzed by fermenting cells of *Acetobacter aceti* MIM 2000/28.^105^ (b) Multistep oxidation of 2-phenylethanol catalyzed by immobilized cells of *Gluconobacter oxidans* NCIMB 8035 under nongrowth conditions.^107^ (c) Multistep oxidation of limonene catalyzed by fermenting cells of *Pseudomonas putida* GS1.^108^ (d) Multistep oxidation of 1,2-diols catalyzed by cells of *Sphingomonas* sp. HXN-200 under nongrowth conditions.^110^

*Gluconobacter oxidans*, another acetic acid bacterium, is frequently used for the oxidation of alcohols to carboxylic acids, such as to synthesize aliphatic acids with chain length between C4 and C7,^106^ or d-(−)-lactic acid from racemic 1,2-propanediol. An interesting study demonstrated the oxidation of 2-phenylethanol to phenylacetic acid using immobilized *Gluconobacter oxidans*, which showed increased tolerance to 2-phenylethanol and a longer production time compared to free cells (Figure 9b).^107^

Different strains of *Pseudomonas putida* have been employed in the selective sequential oxidation of limonene to perillic acid, a relevant compound for the pharmaceutical industry, with product titers of up to 11 g/L (Figure 9c).^108,109^ *Pseudomonas putida* also catalyzes the oxidation of methyl groups on heteroarenes to yield heteroaromatic carboxylic acids. This reaction was scaled up to 1000 L, reaching product titers up to 24 g/L.^100^ Other notable examples are the use of *Sphingomonas* sp. HXN-200 cells to catalyze the regio- and stereoselective oxidations of 3-*O*-benzylglycerol to the corresponding (*R*)-hydroxy carboxylic acid (Figure 9d),^110^ or *Corynebacterium* sp. catalyzing the oxidation of trimethylpropane to 2,2-bis­(hydroxymethyl)­butyric acid. Examples of whole-cell biocatalysts belonging to the fungal kingdom have also been reported, like the use of *Candida tropicalis* cells to convert *n*-alkanes to the corresponding α,ω-dicarboxylic acids.^111^

Oxidation methods applying wild-type whole cells allow the conversion of several primary alcohols under mild conditions, exploiting the enzymes and cofactors already present within the wild-type catalyst. Low production cost of the biomass and the absence of GMO regulations for wild-type microorganisms represent advantages. In general, little prior knowledge of the biological system is required, making the process easy to implement; however, controlling the outcome is usually complex, as the whole metabolism is not optimized for exclusive alcohol oxidation. Consequently, side reactions, low to moderate substrate loadings, and mass transfer limitations through the cell membrane may be issues. Furthermore, the wild-type enzymes are typically expressed at low levels in native strains, and the substrate scope is usually narrow.

### Dehydrogenases

Combining alcohol dehydrogenases (ADH) and aldehyde dehydrogenases (AlDH) in a cascade^112–115^ is commonly adopted for the oxidation of primary alcohols to the corresponding carboxylic acids (Figure 10). In contrast to the wild-type whole-cell approach, defined enzymes are utilized in cell-free systems or as whole-cell catalysts. Both the ADH and AlDH rely on the oxidized nicotinamide cofactors (NAD^+^ or NADP^+^), which are reduced during the oxidation of the alcohol and the aldehyde substrates. To avoid the use of stoichiometric amounts of NAD­(P)^+^, regeneration systems are usually implemented. Depending on the cofactor regeneration system, the stoichiometric oxidant can be either O_2_,^116^ or organic molecules, e.g., α-ketoglutarate (α-KG).^117^ Since ADHs catalyze alcohol oxidation and some ADHs also aldehyde oxidation, different cascade designs are feasible, e.g., utilizing a single ADH for both steps or combining an ADH with an AlDH.

**Figure 10:**
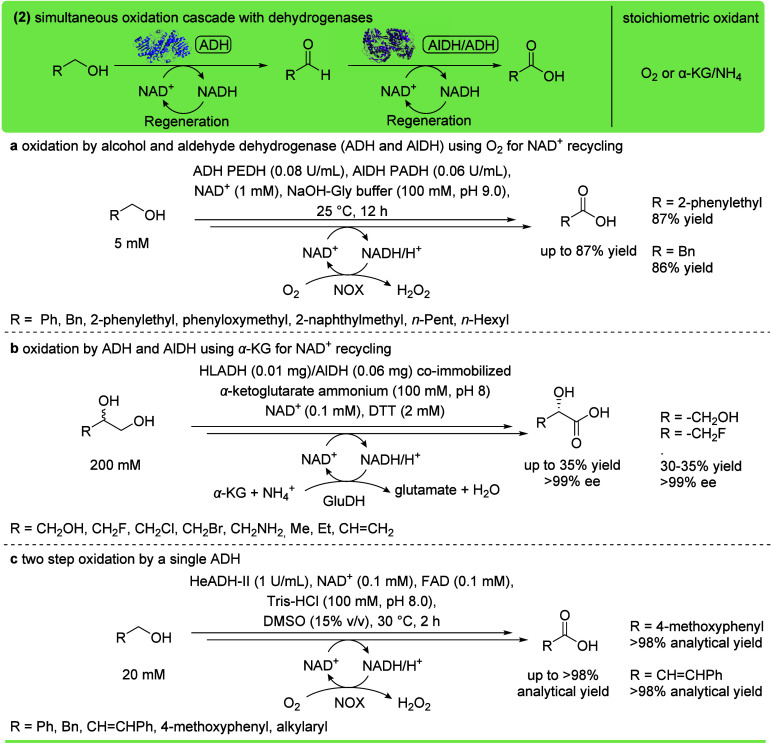
Oxidation of primary alcohols to the corresponding carboxylic acid using alcohol dehydrogenases (ADH, PDB ID 1A4U) and, in selected cases, simultaneously aldehyde dehydrogenases (AlDH, PDB ID 8YEN). (a) Simultaneous oxidation cascade catalyzed by the ADH PEDH and AlDH PADH. NAD^+^ was regenerated with NADH oxidase (NOX).^116^ (b) Oxidation cascade of 1,2-diols catalyzed by horse liver ADH (HLADH) and an AlDH. NAD^+^ was regenerated with glutamate dehydrogenase (GluDH).^117^ (c) Two-step oxidation catalyzed by a single ADH (HeADH-II).^86^

An example of an ADH and AlDH cascade was reported, transforming aromatic and aliphatic alcohols to the corresponding acids using the NADH oxidase (NOX) for NAD^+^ recycling at the expense of O_2_ and simultaneous formation of H_2_O_2_ (Figure 10a).^116^

In a similar cascade, the enantioselective oxidation of 1,2-diols to l-α-hydroxy acids was achieved using coimmobilized ADH and AlDH.^117^ In this case, the NAD^+^ regeneration was performed using a glutamate dehydrogenase, which converted α-ketoglutarate (α-KG) and ammonia to glutamate while consuming NADH (Figure 10b).

In a few cases, a single ADH catalyzed both steps, thus the sequential oxidation of alcohols to aldehyde and finally to carboxylic acid. For instance, the alcohol dehydrogenase HeADH-II catalyzed the oxidation of aromatic or alkyl alcohols, showing high tolerance toward polar solvents and high salt concentrations (Figure 10c).^86^ Furthermore, a lanthanide-dependent ADH (PedH) performed several oxidative steps, converting hydroxymethylfurfural (HMF) to furandicarboxylic acid (FDCA).^118^ Interestingly, two ADHs were also combined for transforming 1,ω-diols. Here, the two ADHs catalyzed diol and lactol oxidation to form a lactone, which was finally hydrolyzed by a lactonase to yield the corresponding ω-hydroxy carboxylic acid (20 mM diol, up to 90% yield).^119^

The oxidation of HMF to FDCA was also performed in a cascade combining a galactose oxidase for alcohol oxidation using O_2_ as oxidant (see the next section) and an ADH for aldehyde oxidation.^120^ Interestingly, the NAD­(P)^+^ cofactor regeneration was achieved through a horseradish peroxidase (HRP), which oxidized the reduced nicotinamide cofactor at the expense of hydrogen peroxide formed by the oxidase, thus scavenging this harmful oxidant that could otherwise cause enzyme inactivation.

Various studies showed the possibility of generating the primary alcohol in situ by hydroxylating alkanes and subsequently oxidizing the alcohol to the corresponding carboxylic acid using ADH and AlDH. For example, the synthesis of tulipalin A was achieved starting with the terminal hydroxylation of isoprenyl acetate mediated by the alkane monooxygenase AlkB, followed by the oxidation with an ADH and an AlDH.^121^ Similar examples used cytochrome P450 monooxygenases,^122,123^ non-heme diiron monooxygenases,^124^ or unspecific peroxygenases^125^ to catalyze the initial hydroxylation of the substrate to generate the primary alcohol.

Overall, dehydrogenases are widely available and well-established biocatalysts for the oxidation of primary alcohols to carboxylic acids. Heterologous expression in *Escherichia coli* is straightforward, and protein engineering is a powerful tool to improve their activity.^118^ The need of nicotinamide cofactors and their recycling make them more complex than some of the following options.^116,120^

### Monooxygenases and Oxidases

Another strategy to access carboxylic acids via a two-step biocatalytic oxidation of primary alcohols involves monooxygenases and oxidases, both utilizing molecular oxygen as a stoichiometric oxidant. Monooxygenases require O_2_ and additionally a reducing equivalent, in general in the form of NAD­(P)­H, which is ideally recycled.^88,126^ While monooxygenases generate water as coproducts, oxidases produce hydrogen peroxide. Each class of enzyme can potentially catalyze the oxidation of the alcohol as well as the oxidation of the aldehyde hydrate.

For the electron transfer from NAD­(P)H to the iron center of monooxygenases, a complex machinery is operating, involving, for instance, electron carrier proteins like ferredoxin, ferredoxin reductases, or cytochrome P450 reductases.^127^ Due to this complexity and the need for a reducing agent to recycle the NAD­(P)­H, sacrificial electron donors are usually added, such as sugars and other carbohydrates (e.g., glycerol) for whole-cell systems.^128,129^ Nevertheless, stoichiometric amounts of nicotinamide cofactors in the case of cell-free biocatalysis have also been described.^130^ Due to this complexity, whole-cell biocatalysts are the preferred format for this approach, since the monooxygenase and the electron carrier proteins are simultaneously expressed in a heterologous host.^131,132^

Two classes of monooxygenases were identified as capable of catalyzing the reaction of interest: cytochrome P450 monooxygenases (CYP450s), which use a heme prosthetic group, and non-heme diiron monooxygenases, which feature two iron atoms in their active site. CYP450s were employed in several studies, such as the in vitro conversion of ethanol to acetic acid by CYP2E1,^130^ or the 3-step oxidation of sterols by the sterol-27-hydroxylase (a mitochondrial CYP450) expressed in mammalian cells.^133^

In other cases, plant CYP450s, like CYP94A5, were expressed in yeast, which catalyzed the terminal oxidation of fatty acids to dicarboxylic acids,^134^ or CYP71AV1, used to synthesize the antimalarial drug precursor artemisinic acid starting from the sesquiterpene amorpha-4,11-diene.^135^ Bacteria belonging to the actinomycetes are an important source of CYP450s, of which many are involved in the biosynthesis of natural products.^136^ For instance, the CYP450 RosC was coexpressed in *E. coli* together with the electron carrier proteins CamA (putidaredoxin) and CamB (putidaredoxin reductase). The whole-cell catalyst catalyzed the multistep oxidation of 20-dihydrorosamicin to 20-carboxyrosamicin (Figure 11a).^137^

**Figure 11:**
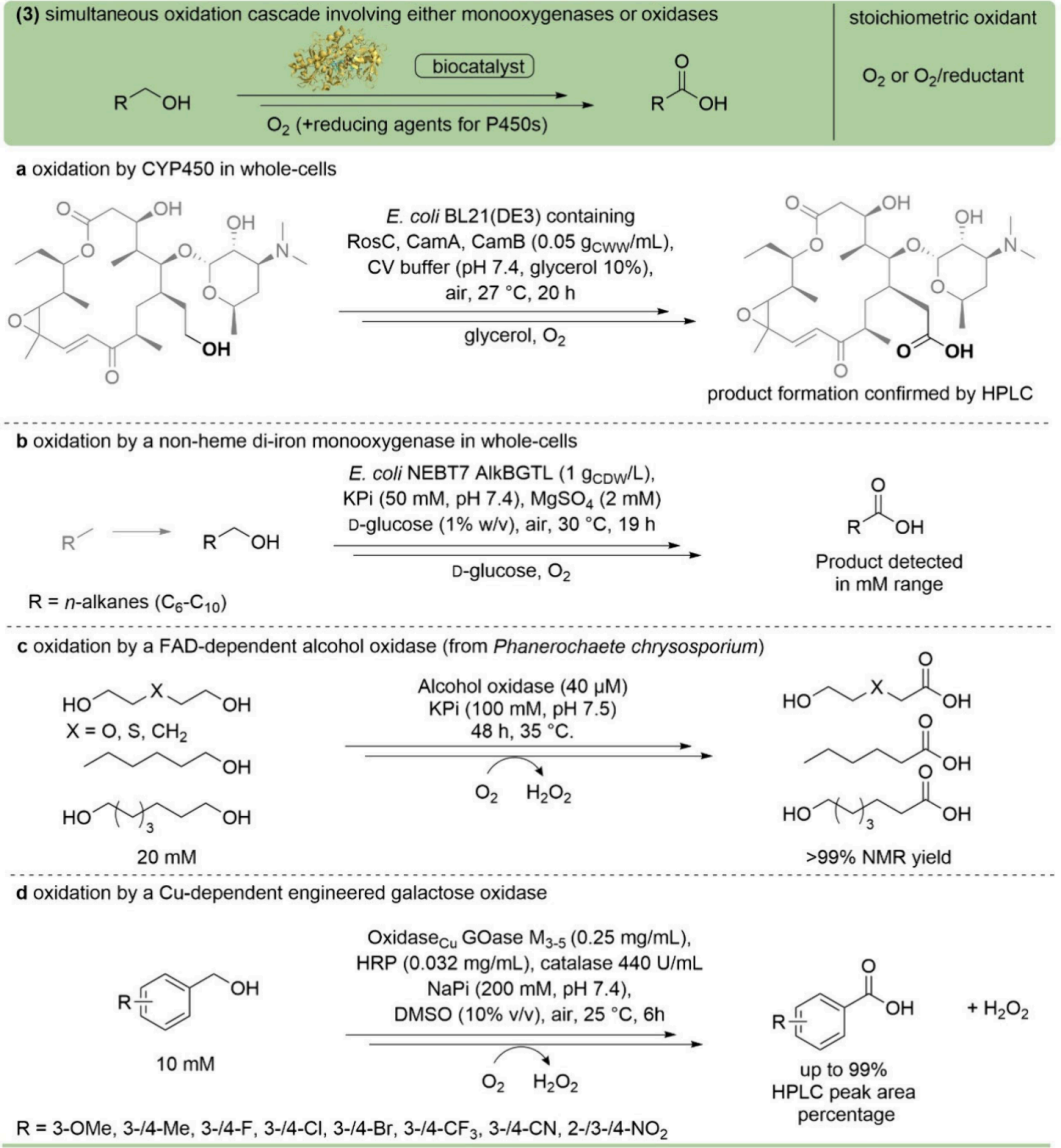
Two-step oxidation of primary alcohols to acids employing monooxygenases (cytochrome P450 monooxygenases, or nonheme diiron monooxygenases) or oxidases (PDB ID 4MJW) using O_2_ as a stoichiometric oxidant. (a) Oxidation of 20-dihydrorosamycin catalyzed by the CYP450 RosC coexpressed in whole cells (*E. coli*).^137^ (b) Oxidation of *n*-alkanes catalyzed by the non-heme diiron monooxygenase AlkB (using whole *E. coli* cells).^140^ (c) Multistep oxidation of 1,ω-diol and 1-hexanol to carboxylic acids catalyzed by the FAD-dependent alcohol oxidase.^147^ (d) Multistep oxidation of primary alcohols catalyzed by the engineered Cu-dependent oxidase Goase M_3–5_.^148^

The following synthetic applications to access carboxylic acids involve non-heme diiron enzymes. The multistep oxidation of toluenes or xylenes was catalyzed by xylene monooxygenase (XylM) expressed in *E. coli* together with the reductase XylA,^138^ while the conversion of alkanes to mono or dicarboxylic acids was enabled by AlkB, coexpressed in *E. coli* with AlkG and AlkT as electron carrier proteins (Figure 11b).^139,140^

Oxidases are stand-alone enzymes requiring molecular oxygen for their activity and generating hydrogen peroxide as a coproduct.^97,98,141–143^ Consequently, catalases are often added to disproportionate the harmful peroxide into water and O_2_.^144^

Among flavin-dependent oxidases, hydroxymethylfurfural oxidase (HMFO) was used to catalyze the multistep oxidation of HMF (5 mM) to the corresponding dicarboxylic acid FDCA (95% product, TON: 570).^145^ Moreover, rational engineering of the active site of HMFO enhanced the oxidation of aldehyde intermediates, allowing the conversion of several substituted benzylic alcohols to the corresponding acids, albeit with a low percentage of acid formation.^146^ Another example is the alcohol oxidase AOX from *Phanerochaete chrysosporium*, which catalyzes the multistep oxidation of aliphatic primary alcohols and 1,ω-diols to the corresponding acids and hydroxyacids or oxa acids, respectively, with a TON of up to 1500 (Figure 11c).^147^ Furthermore, the choline oxidase, also flavin-dependent, was engineered to increase its thermal and solvent stability, as well as the substrate scope, leading preferentially to the aldehyde.^82^ Nevertheless, overoxidation was observed for cinnamyl alcohol to the corresponding acid at 20% conversion.

In addition to flavin-dependent oxidases, the galactose oxidase GOase is a copper-dependent enzyme, which was engineered (Goase M_3–5_ mutant) to oxidize different substituted benzylic and heteroaromatic benzylic alcohols (Figure 11d).^148^ Furthermore, the Goase M_3–5_ variant was also used in cascades combined with either a xanthine dehydrogenase (a flavin-dependent oxidase),^149^ or the aldehyde oxidase PaoAC, to achieve the conversion of HMF to FDCA, as well as several aliphatic and aromatic alcohols to the corresponding acids.^150^

Transformations with monooxygenases are, in general, considered challenging due to the complexity of the electron transfer system. At a first glance, oxidases seem to be a highly interesting option, but substrate loadings are still low, as well as turnover numbers. As only a few examples have been published until now, progress may be expected in the future.

### Peroxygenases

Since their discovery in 2004,^151^ unspecific peroxygenases (UPOs), being heme-dependent enzymes, have gained increasing attention due to their ability to efficiently accept H_2_O_2_ as oxidant as well as to their broad substrate scope. Furthermore, UPOs show impressive stability in the presence of H_2_O_2_, making them a more viable option compared to O_2_/NAD­(P)­H-dependent P450s.^152,90^ Consequently, unlike CYP450s, peroxygenases do not need additional proteins to shuttle electrons or sacrificial electron donors. UPOs have been used for the sequential oxidation of HMF to FDCA catalyzed by *Mor*UPO and *Aae*UPO, in a two-step one-pot process, achieving 95% conversion of 100 mM substrate (Figure 12a).^153^ Due to high substrate loading and the low enzyme concentration, TONs reached up to 13500.

**Figure 12:**
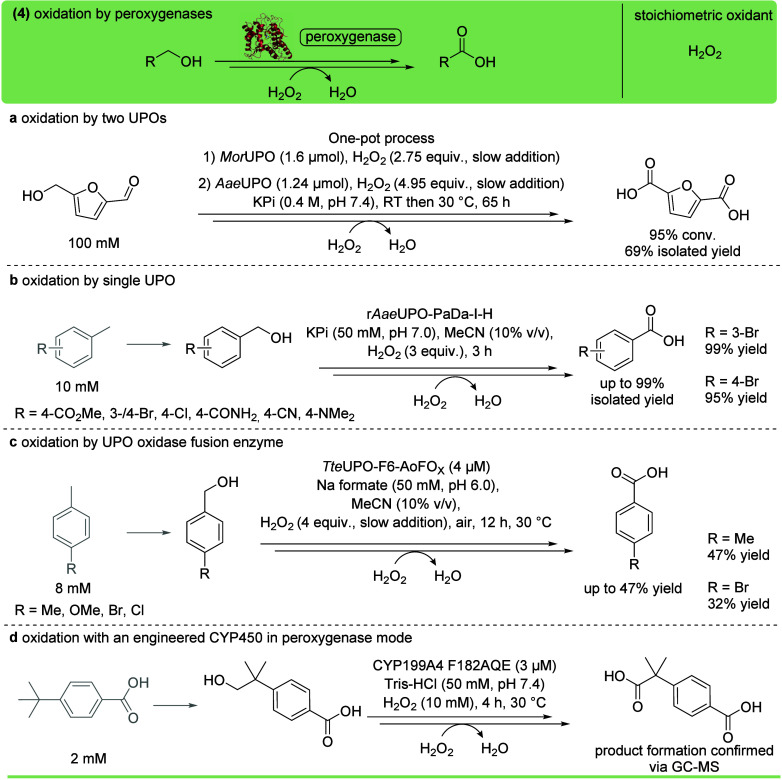
Oxidation of primary alcohols with heme-containing enzymes, including unspecific peroxygenases (UPOs, PDB ID 9J1Q) or cytochrome P450 monooxygenases in peroxygenase mode using H_2_O_2_ as a stoichiometric oxidant. (a) Multistep oxidation of HMF catalyzed by *Mor*UPO and *Aae*UPO in a two-step, one-pot reaction.^153^ (b) Multistep oxidation of toluene derivatives catalyzed by r*Aae*UPO-PaDa-I-H.^154^ (c) Multistep oxidation of toluene derivatives catalyzed by *Tte*UPO fused with the formate oxidase *Ao*FO_*x*_.^83^ (d) Multistep oxidation of 4-*tert*-butylbenzoic acid catalyzed by engineered CYP199A4 in peroxygenase mode.^155^

A single UPO, namely the PaDa-I variant of the UPO from *Agrocybe aegerita* (r*Aae*UPO-PaDa-I-H), was used to catalyze the oxidation of the terpenoid 3-carene to chaminic acid,^156^ and of different toluenes and benzylic alcohols to the corresponding acids (Figure 12b).^154^ The same enzyme also showed the ability to oxidize (*E*)-allylic alcohols selectively from an *E*/*Z* mixture to the carboxylic acid/aldehyde products, respectively.^157^

A fusion protein of *Tte*UPO with the formate oxidase AoFOx was used for the oxidation of toluene derivatives, yielding the corresponding carboxylic acids. In this approach, the H_2_O_2_ required for the activity of *Tte*UPO was generated in situ via the oxidation of formate catalyzed by the formate oxidase. Furthermore, H_2_O_2_ was also provided externally by slow addition for increasing product formation (Figure 12c).^83^

Nevertheless, among heme-dependent enzymes, not only UPOs, but also a few CYP450s were reported to have peroxygenase activity.^158–160^ This feature was observed with CYP199A4, which was engineered to further increase its peroxygenase activity and used to perform the multistep oxidation of 4-*tert*-butylbenzoic acid to a dicarboxylic acid (Figure 12d).^155^

Despite the encouraging advances in UPO-catalyzed oxidations, their applicability still faces some challenges. For instance, the heterologous expression is often poor or not yet feasible in conventional bacterial systems like *E. coli*.^90,161^ This issue is usually solved by using yeast (e.g., *Komagataella phaffii*) as a heterologous expression host, which excretes the enzymes into the medium, allowing straightforward enzyme isolation.^90^ Furthermore, UPOs are, like any enzyme, prone to deactivation by H_2_O_2_. To avoid/minimize degradation, the reactive oxidant is either generated in situ,^144,162,163^ or constantly fed to keep its concentration at a tolerated level.^153,164^

## Future Perspectives

The perspective shows that oxidation methods to transform primary alcohols to the corresponding carboxylic acids have evolved over time. The field has progressed from “brute force” methods relying on metalates in (over)­stoichiometric amounts over noncharacterized whole-cell oxidations, to finely tuned catalytic approaches, utilizing environmentally benign oxidants such as H_2_O_2_ or molecular oxygen. This development can be observed for both chemical catalysts and biocatalysts. While dehydrogenation methods have been frequently reported for chemical systems, this has not been shown for biocatalysts, yet. However, recent research^165^ suggests that biocatalytic transfer-dehydrogenation of alcohols to acids could be realized by exploiting, for instance, a hydrogenase that recycles NAD^+^ from NADH, releasing H_2_ as the sole byproduct. Similarly, the oxidation of alcohols to acids has been performed using electrochemical methods, but comparable enzymatic strategies remain undeveloped, although they appear feasible based on advances in related biocatalytic reactions.^166–168^

Considering the reported data for the oxidation of primary alcohols to the corresponding acids, chemical catalysts show turnover numbers mostly in the range of 100, sometimes reaching up to 10000. Comparable performance has been observed for the enzymatic approaches, where TONs up to 13500 were reached for the oxidation of alcohols to acids employing UPOs.^153^ In general, the trend is heading toward methods with low environmental impact, which is expected to go hand in hand with reduced costs. The simpler the reagent (O_2_, H_2_O_2_, or even no oxidant in case of dehydrogenation), the fewer coproducts are formed, and the less waste needs to be considered. Factors such as the amount and the type of solvent, reaction temperature, and the catalyst also influence the overall environmental footprint. To reduce process costs and enhance recyclability and biodegradability, catalysts should be readily manufactured from renewable sources, exhibit high stability, and deliver high total turnover numbers (TTN), thus the TON during the lifetime of the catalyst. While for pharma products TONs of 4000 are acceptable, specialty chemicals require a TON of around 80,000.^13,169^ Future advances will particularly depend on increasing the turnover numbers (TON) for all types of catalysts, accompanied by improving stability and activity; for enzyme, e.g., via protein engineering or de novo enzyme design.^79–83^ Furthermore, to reach high space-time yields an elevated substrate concentration is important. Chemical methods usually operate at higher substrate concentrations (>100 mM) compared to biocatalytic methods, which need to be improved in the future to reach competitive substrate loadings of at least 50–100 mM.

## Abbreviations

α-KG: α-ketoglutaric acid
ABTS: 2,2′-azinobis­(3-ethylbenzothiazoline-6-sulfonic acid)
ACT: 4-acetamido-TEMPO
ADH: alcohol dehydrogenase
AlDH: aldehyde dehydrogenase
CYP450: cytochrome P450 monooxygenase
DCE: 1,2-dichloroethane
eq. or equiv: equivalents
DMF: dimethylformamide
FADH_2_: flavin adenine dinucleotide, reduced form
FDCA: furandicarboxylic acid
GMO: genetically modified organism
GMP: Good Manufacturing Practice
HMF: hydroxymethylfurfural
HRP: horseradish peroxidase
LED: light-emitting diode
NAD­(P)­H: nicotinamide dinucleotide (phosphate)
NMO: *N*-methylmorpholine *N*-oxide
NOX: NADH oxidase
NSAID: nonsteroidal anti-inflammatory drug
PDB: Protein Data Bank
PDC: pyridinium dichromate
PET: polyethylene terephthalate
PTC: phase transfer catalyst
Salen: *N*,*N*′-bis­(salicylidene)­ethylenediamine
TEMPO: 2,2,6,6-tetramethylpiperidin-1-oxyl
TON: turnover number
TPAP: tetra-*n*-propylammonium perruthenate
TPPT: 2,4,6-triphenylpyrylium tetrafluoroborate
TTN: total turnover number
UPO: unspecific peroxygenase
Δ*G*: Gibbs free energy change
